# Differentiation of canine distemper virus isolates in fur animals from various vaccine strains by reverse transcription-polymerase chain reaction-restriction fragment length polymorphism according to phylogenetic relations in china

**DOI:** 10.1186/1743-422X-8-85

**Published:** 2011-02-27

**Authors:** Fengxue Wang, Xijun Yan, Xiuli Chai, Hailing Zhang, Jianjun Zhao, Yongjun Wen, Wei Wu

**Affiliations:** 1 Institute of Special Economic Animals and Plants Science, The Chinese Academy of Agricultural Sciences, Jilin 132109, People's Republic of China; 2 Jilin Zhongte Biotechnological Co. Ltd, Jilin 132109, People's Republic of China

## Abstract

In order to effectively identify the vaccine and field strains of Canine distemper virus (CDV), a new differential diagnostic test has been developed based on reverse transcription-polymerase chain reaction (RT-PCR) and restriction fragment length polymorphism (RFLP). We selected an 829 bp fragment of the nucleoprotein (N) gene of CDV. By RFLP analysis using *Bam*HI, field isolates were distinguishable from the vaccine strains. Two fragments were obtained from the vaccine strains by RT-PCR-RFLP analysis while three were observed in the field strains. An 829 nucleotide region of the CDV N gene was analyzed in 19 CDV field strains isolated from minks, raccoon dogs and foxes in China between 2005 and 2007. The results suggest this method is precise, accurate and efficient. It was also determined that three different genotypes exist in CDV field strains in fur animal herds of the north of China, most of which belong to Asian type. Mutated field strains, JSY06-R1, JSY06-R2 and JDH07-F1 also exist in Northern China, but are most closely related to the standard virulent strain A75/17, designated in Arctic and America-2 genetype in the present study, respectively.

## Background

Canine distemper virus (CDV) is an enveloped negative-strand RNA virus classified into the genus Morbillivirus within the family Paramyxoviridae. CDV has a very broad host range, having been isolated from dogs, red pandas, foxes, raccoon dogs and tigers, and also identified in giant pandas, lions, lynxes, golden cats, bears and wolves [1-4]. Since 1972, the number of clinical cases of distemper in dogs and fur animals increased rapidly, when large mink and fox farms were established in China. CDV infection results in systemic disease with involvement of the central nervous system and the respiratory and gastrointestinal tracts [5]. The economic loss is significant due to CDV infection.

Although vaccination against CDV with attenuated virus can protect the majority of animals, this protection does not necessarily extend to the field strains. The accurate diagnosis of CDV infection can be made with the use of methods based on molecular biological techniques. In recent years, the application of nucleic acid hybridization, PCR and other techniques[6-9] has improved the accuracy, sensitivity and specificity of CDV diagnosis. The detection of CDV based on N gene has been established by many researchers [10-14]. Recently, real-time RT-PCR targeting the hypervariable C-terminal domain of the nucleocapsid (N) gene was established and shown to be more sensitive and effective [15]. The use of RT-PCR-RFLP to detect the hemagglutinin (H) gene of CDV was reported by Calderon [16]. However, the CDV N gene is more conserved than H gene, and therefore, provides a better target for CDV detection.

Identification of vaccine and field strains of CDV is very important for control the disease. The contagious nature and the high mortality rates of canine distemper make it necessary to speed up the diagnostic procedure in order to quarantine infected animals and start appropriate treatments early. CDV is a highly effective prophylactic. Accurate vaccination and good surveillance programs are of great importance, and reliable detection methods are needed. So the tool of differentiation of vaccine and field strains is the first step to do. In this study, an RT-PCR-RFLP method based on CDV N gene was established, which could effectively differentiate the vaccine and field strains of CDV. This method not only met the need for veterinary technology in the fur industry, but also supplied a theoretical foundation for field strain genotyping and furthered the basic research of CDV.

## Methods

### Viral strains, cells and samples

The vaccine strain, CDV_3_, which has been most widely used in the prevention of canine distemper in domestic fur animals, was identified and preserved by the Zoonosis Lab, Institute of Special Field Economic Animal and Plant Science, the Chinese Academy of Agricultural Sciences. The commercial vaccine products included vaccine 1: INTERVET DHPPI (Holland), vaccine 2: VIRBAC hexade vaccine (France), vaccine 3: Norden Chimeric quadruple vaccine (USA) and vaccine 4: Laboratories HIPRA septuple vaccine (Spain). All the N gene segments of CDV in these combinative vaccines of the present study were respectively labeled as vaccine 1, vaccine 2, vaccine 3, vaccine 4. African green monkey kidney cells were purchased from China Institute of Veterinary Drug Control. The blood or tissue samples from infected animals were collected from breeding fields in different provinces of northeast China.

### Primers

GenBank sequence analysis revealed a *Bam*HI site at position 1086 in the genome of CDV field strains [GenBank: AY443350, GenBank: AY649446, GenBank: AY445077, GenBank: AY542312, GenBank: AY466011, GenBank: AY386315, GenBank: AY386316] and the standard virus strain (A75/17) but not in the vaccine strain (Onderstpoort). After analysis of the N gene in other field isolates [GenBank: AY390348, GenBank: AJ009656, GenBank: DQ887066, GenBank: DQ003302, GenBank: EF445056, GenBank: DQ435615, GenBank: EF445050, GenBank: DQ522030] and the vaccine strain [GenBank: EF418783], a difference in the *Bam*HI site was observed. Therefore, this could serve as a valuable tool in the differentiation of field strains from vaccine strains.

A conserved segment (372-1200 bp) of CDV N gene (Onderstepoort) was selected and primers designed (P1: 5'TTCTG AGGCA GATGA GTTCT TC 3', P2: 5'CTTGG ATGCT ATTTC TGACA CT 3'), with a product length of 829 bp. The primer set was synthesized by Shanghai Yingjun biotechnological Co. Ltd (Invitrogen, Beijing, China).

### Preparation of the viral RNA

The tissues and blood of infected animals were collected using sterile technique. The tissues were ground after adding 1:1 PBS (weight: volume). The total RNA was extracted using the RNAeasy kit (QIAGEN) according to the manufacture's instructions. Total RNA of virus culture was extracted with TRIzol (Invitrogen) and eluted using DEPC (diethyl pyrocarbonate)-treated water. The RNA was either directly used in RT-PCR or preserved at -70°C for later use.

### RT-PCR

The PCR amplication cycle was optimized as follows: 94°C 45 s, 52.2°C 45 s and 72°C 45 s, for 35 cycles with a final extension step at 72°C for 5 min. Five microliters of the amplified products were visualized using gel electrophoresis in a 1.0% agarose gel in the presence of ethidium bromide. Bands were visualized by ultraviolet light transillumination and compared with a 100 bp Ladder (TakaRa).

### Recovery, purification and RFLP analysis on PCR products

The positive bands were purified by gel extraction (TIANGEN) and subsequently used for the RFLP experiment. The RFLP reaction was performed as follows. Two μl 10× buffer, 1μl *Bam*HI (TaKaRa), 6μl PCR product and 11μl aseptic deionized water were combined into 20 ul total reaction volume. The reaction was then incubated in 37°C water bath for 1-1.5 h, after which time, 2 μl of 10× Loading buffer was added. The results of agarose gel electrophoresis were used to determine the type of virus in the sample.

### Sequencing and genetic analysis of N gene of isolated CDV field virus strains

To confirm the occurrence of the target gene sequence and *Bam*HI site, all of the amplified segments were cloned into the pMD18-T vector (TaKaRa) and sequenced at Shanghai Yingjun Biotechnological Co. Ltd (Invitrogen, Beijing, China). The sequencing result was analyzed and 829 bp segments of 19 field strains were compared to cognate sequences of a selection of 21 CDV strains of various animal and geographical origins and 5 vaccine strains. Phylogenetic analysis was performed.

## Results

### Amplification and purification of the target gene segments

The total RNA was extracted from suspected CDV samples and vaccine strain. The target segment was amplified under optimized conditions. A specific 829 bp fragment of N gene was obtained from 19 CDV field strains and 5 CDV attenuated strains contained in the commercial vaccines (data not shown). The positive products were purified by gel extraction and stored at -20°C for later use.

### RFLP analysis

**Figure 1:**
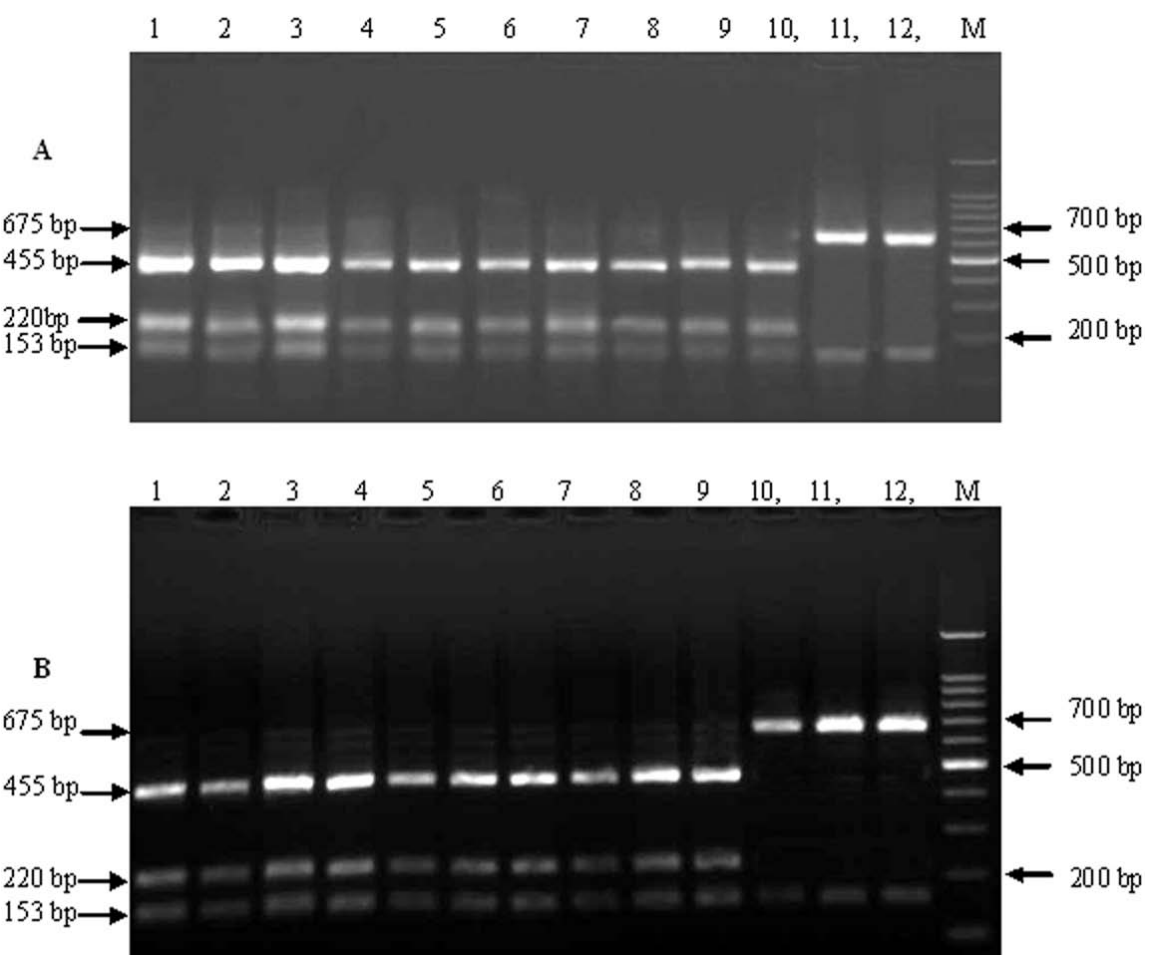
**RFLP electrophoresis result to identify the amplified 829 bp segments of CDV vaccine strains and field strains**. A) Lane 1-10: LDL05-M1, LDL05-F1, SDWD06-M1, LHLD06-M1, LHC06-F1, JSY06-R1, LLY06-M1, SN07-R1, HCL07-R1, LHS07-R1; Line 11,12: CDV3 and Spanish; M: 100 bp Ladder DNA Marker; B) Lanes 1-9: JDH07-F1, JDAN07-R1, LDL05-M2, LDL05-F2, SDWD06-M2, LHLD06-M2, JSY06-R2, HSN07-R2, HCL07-R2; Lanes 10-12: France, Holland and USA; M: 100 bp Ladder DNA Marker. Three bands were seen in Figure 1A(Lanes 1-10) and Figure 1B(Lanes 1-9): 455 bp, 220 bp and 153 bp, which belong to CDV field strains; two bands were showed in Figure 1A(Lanes 11,12) and Figure 1B(Lanes 10-12): 675 bp and 153 bp, which are CDV vaccines.

RFLP analysis was carried out on the purified target segments and the results were used to identify the corresponding virus as vaccine or field strain. According to the analysis, the 829 bp PCR product was cleaved into two segments by *Bam*HI: 675 bp and 153 bp in the vaccine strains. The field strains were cleaved into three segments: 455 bp, 220 bp and153 bp (Figure 1). Lanes 1-10 in Figure 1A and lanes 1-9 in Figure 1B show the RFLP results of the amplified 829 bp segment in the 19 samples from clinically suspected canine distemper animals. These strains are from several provinces: Hebei, Shandong, Liaoning and Jilin. They are isolated in minks, raccoon dogs and foxes. Lanes 11, 12 in Figure 1A and lanes 10-12 in Figure 1B show the RT-PCR-RFLP results of vaccine strains, which are CDV_3_, Spanish, France, Holland and USA. From the figure, the 829 bp segments amplified from suspected CDV animals could all be digested by *Bam*HI into three fragments, suggesting the detected CDV strains in these tissues are field strains; while the vaccine strains show the theoretical 2 fragment pattern.

### Sequencing and phylogenetic analysis of the isolated field virus strains

**Figure 2:**
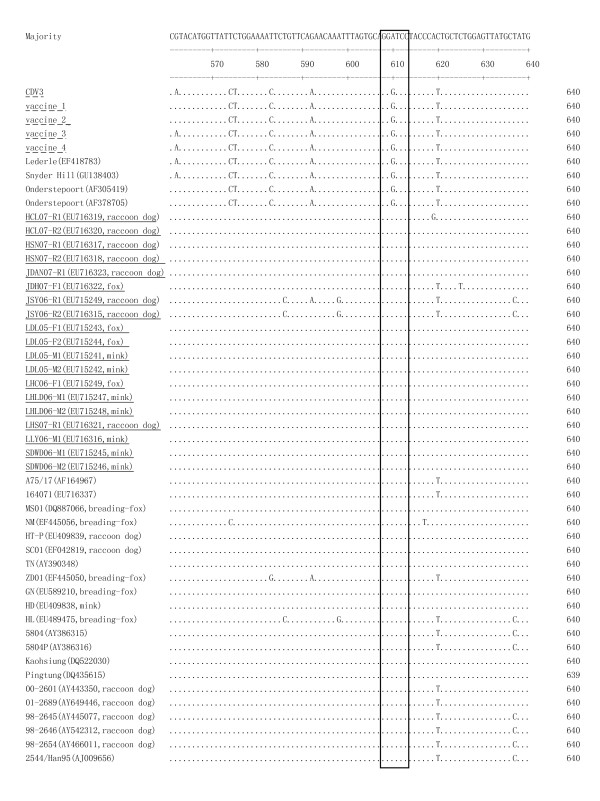
**A single nucleotide difference leads to the existence of an addition *Bam*HI site of CDV field strains**. All CDV isolates have an A at 610 nt in N gene 829 bp fragments, but vaccine strains have a G. (*Bam*HI site GGATCC showed in box). Underline indicates the 19 Chinese CDV field strains analyzed in this study. Underline with broken line means commercial CDV vaccines analyzed in this study.

The PCR product of the putative field strains were sequenced and submitted to GenBank. The 610^th ^base of the 829 bp segment (correlating to the 981^st ^base in the total N gene) in field strains was different from that in vaccine strains. It was the conversion of the 610^th ^base from G to A leading to an additional *Bam*HI site in 829 bp segment of N gene in field CDV strains compared to CDV vaccine strains (Figure 2). Thus there are 2 *Bam*HI digestion sites in the 829 bp segment of the N gene in CDV field strains, whereas only one in vaccine strains (sequence of the five CDV commercial vaccine). Additionally, this single nucleotide differentiation is observed between 3 CDV vaccine strains (Lederle, Snyder Hill, Onderstepoort) and 21 CDV isolates in the GenBank sequences, indicating the *Bam*HI site can distinguish these CDV vaccine strains and field strains.

**Figure 3:**
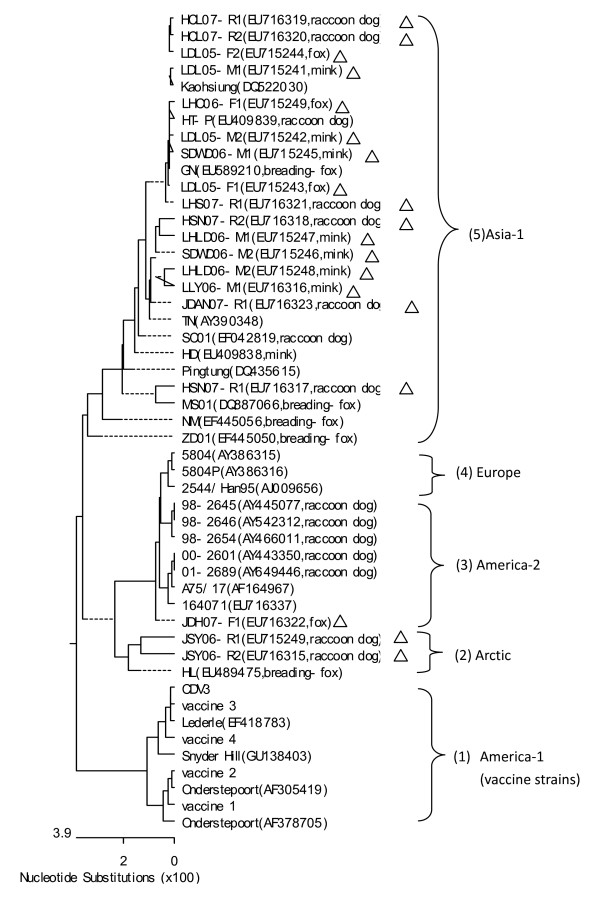
**Phylogenetic analysis of the N gene of field CDV strains**. (1) vaccine strains, or America-1 genotype; (2) (3) (4) (5) four branches of the CDV field virus strains, (2) The branch belonging to Arctic genotype. Two strains (JSY06-R1 and JSY06-R2) of CDV isolated from Jinlin Songyuan raccoon dog, distinguished from other strains, are similar to HL, and were clustered into Arctic genotype; (3) America-2 CDVs genotype. JDH07-F1, isolated from Jilin Dehui slivery fox, which was most closely related to the standard field strains A75/17, which belong to America-2 CDVs; (4) Europe genotype. This cluster has not been identified in China. (5) Asia-1 genotype. The 16 field strains in present study are closely related to many field strains, which from China mainland or Taiwan China, and belong to Asia-1 genotype. Δ means CDV isolates in this study.

Furthermore, the nucleotide sequences of the RT-PCR products from 19 field isolated strains and CDV of in the five commercial vaccine were analyzed. The 829 bp nucleotide fragments of the N gene were sequenced and then aligned with corresponding sequences of the CDV strains obtained from the GenBank database. Figure 3 shows the distance-based Neighbor-Joining tree displaying the phylogenetic relationship among 49 CDV strains based on one part of N gene. In the phylogenetic tree, most of the Chinese field strain (16/19) grouped together in one branch in the Asia-1 genotype. However, three strains (JSY06-R1 and JSY06-R2, isolated from raccoon dog in Jilin Songyuan, and JDH07-F1 from Jilin Dehui silvery fox) obviously belong to distinct branchs. The raccoon dog strains JSY06-R1 and JSY06-R2 displayed the highest identity to HL, a fox strain identified from Heilongjiang province and were designed in Arctic genotype. The fox strain JDH07-F1 has the highest homology with the standard virulent strain A75/17, which is belong to America-2 genotype. The other 16 field strains are similar to published strains on the China mainland, such as HD, TN, MS01 and PingTung in the genotype of Asia-1.

## Discussions

Due to a worldwide distribution and the broad host-range of CDV including a vast wildlife reservoir, and available in large amounts of unrelated species of animals in interspecific spreads, eliminate CDV is unlikely, therefore, prevention of disease is very important. Timely diagnosis, differential detection of strain type of CDV, reasonable utilization of vaccine to prevent the disease effectively, can obtain the effective control of canine distemper. So the method of differentiation of CDV vaccine and field strains is needed to construct. Nucleocapsid protein (N) gene is most conserved structural proteins gene of CDV.

Sidhu et al have found the 981^st ^base of N gene originating from some CDV vaccines has changed from G to A in the field strains [17]. This leads an additional *Bam*HI site, which allows for the clinical discrimination. During sequence alignment of the CDV genome, a difference between CDV field strains and vaccine strains was observed and therefore a method developed to identify them.

In present study, we report on the development of RT-PCR-RFLP suitable for the rapid and sensitive discrimination of CDV field strains and vaccine strains. The assay is based on the conserved CDV N gene. An 829 bp segment between 372 bp and 1200 bp of the CDV N gene was amplified and used in RFLP analysis. By the result banding pattern, one could identify the strain as vaccine or field. After digestion by *Bam*HI, electrophoresis showed two fragments (659 and 153 bp) in the vaccine strain and three fragments (455, 220 and 153 bp) in the field strains. Due to fur animal breeding at present mainly concentrated in north and northeast of China, we collected several CDV vaccine on the domestic and international markets and animal organs suspected happening distemper in north and northeast of China. The collected tissues were investigated including 19/83 RT-PCR positive (data not shown).

When Calderon characterized CDV from vaccinated and non-vaccinated dogs, he found that the CDV H gene RFLP analysis by *Nhe*I could identify vaccine and field virus strains of CDV [16]. But CDV H gene had more variable than N gene [18-20]. This novel method (RT-PCR-RFLP) to identify vaccine and field virus strains of CDV targets the conserved N gene and the amplified segment (829 bp) is easy to manipulate. Confirmation of field virus infection (or vaccine virus artifact) animals in the clinic could be completed in 6 hours. Five CDV vaccines on the market, including vaccines from China, Holland, France, USA, and Spain, were analyzed, and only 2 fragments by the 829 bp fragment of CDV N gene RFLP analysis were observed, coinciding with one *Bam*HI digestion site. Furthermore, the sequences were identical to the Onderstepoort strain displayed in GenBank [16]. The 19 field isolates coming from northeast and north of China from 2005 to 2007, all displayed 3 fragments and contain 2 *Bam*HI sites, identical to the N gene sequence of CDV isolates in GenBank. Then this new assay is suitable to accurately distinguishing between the CDV vaccine and field strains. The assay is also precise, and efficient and will give an excellent technological support that will in turn cultivate the fur industry in China. To demonstrate the novelty and utility of this new assay, a patent invention (CN200710056023.4) was awarded.

Phylogenetic analysis based on H gene[16,21,22], there are Aisa-1, Aisa-2, Aisa-3, Europe, Europe wild-life, America-2 and Arctic genotype CDV field strains. In this study, the part of CDV N genes of 19 Chinese field CDV strains detected from breeding foxes, raccoons and minks strains were sequenced and analyzed. A genetic analysis of the N gene 829 bp segments of these field CDV strains was performed. Phylogenetic distances suggested that there are three types CDV field strains in fur animals in Northern China. Most of the field CDV strains coming from northeast and north of China from 2005 to 2007 belong to Asia-1 type. Two strains from Jilin (JSY06-R1 and JSY06-R2) must be mentioned, as they are remarkably different from other isolates and presumably have diversity due to gene mutation. In previous report, three different CDV genotypes (Asia-1, Arctic, and Asia-3) were currently circulating in breeding foxes, raccoon dogs and minks in China based on H gene of CDV phylogenetic analysis[22]. Here, strains JSY06-R1 and JSY06-R2, identified from Jilin province in 2006, displayed the highest identity to HL strain, belong to Arctic genotype. Arctic CDVs had been reported since 1980 s [23,24] and then widespread in Europe[25,21,26]. In the late 1990 s, Arctic CDVs arised in China[27]. At present, Arctic CDVs, such as strain HL from Heilongjiang province[22], strain Liu from Jilin province[27] and JSY06-R1 and JSY06-R2 from Jilin province in this study, are all exist in northeast of China.

Asia-3 CDV in breeding foxes, raccoon dogs and minks in China were firstly described in 2010[22]. However, the present study further revealed the existence of America-2 genotypes of CDV in breeding foxes in China. By phylogenetic analysis, strain JDH07-F1 from fox had been found to belong to the branch with classical virulent virus A75/17, which belonged to CDV America-2 genotypes (Figure 3). Wild-type CDV strain A75/17 is a highly virulent strain, which induces a persistent infection in the central nervous system with demyelinating disease[28]. Strain JDH07-F1 was highly identical (99.2% nucleotide) with Wild-type strain A75/17 in the N protein gene. So it was speculated that the virulence of JDH07-F1 strain must be strong. Further study will explore the biological characteristics of these virulent virus strains.

## Conclusions

An ingenious method had been applied to identify vaccine and field virus strains of CDV. RFLP analysis of the 829 bp fragment of CDV N gene can be digested into two fragments(659 and 153 bp) in the vaccine strain and three fragments (455, 220 and 153 bp) in the field strains by *Bam*HI. We analyzed N gene of 49 strains of CDV (including 19 new field isolates coming from northeast and north of China during 2005 and 2007). The results showed the vast majority of the CDV strains analyzed in this study were characterized as Asia-1 genotype. Strain JSY06-R1, JSY06-R2 and JDH07-F1 are belong to Arctic and America-2 type, respectively. And JDH07-F1, a first America-2 type CDV, is close to A75/17, maybe with higher virulence.

## Competing interests

The authors declare that they have no competing interests.

## Authors' contributions

FXW was responsible for experimental design, analyses and drafting of the manuscript, and performed RT-PCR and RFLP assays, and analyze sequence. XLC and HLZ participated in virus culture and virus isolation. JJZ did the work of collection of specimens. XJY and WW were responsible for supervision of the study. YJW review the manuscript and make figures and tables. All authors read and approved the final manuscript.

## Aknowledgements

The study was financially supported by the grant of the Social Common wealth Research Special funds of Scientific Research Institute. The authors thank Dr. Lisa Mc Ewen and Mary Charlotte Guest (College of Veterinary Medicine, University of Georgia, USA) helps in preparation of the manuscript.

## References

[B1] Alexander KA Appel MJ African wild dogs (Lycaon pictus) endangered by a canine distemper epizootic among domestic dogs near the Masai Mara National Reserve, Kenya J Wildl Dis 1994 30 4 481 485 7760475 10.7589/0090-3558-30.4.481

[B2] Cranfield MR Barker IK Mehren KG Rapley WA Canine Distemper in Wild Raccoons (Procyon lotor) at the Metropolitan Toronto Zoo Can Vet J 1984 25 2 63 66 17422359 PMC1790529

[B3] Mainka SA Qiu X He T Appel MJ Serologic survey of giant pandas (Ailuropoda melanoleuca), and domestic dogs and cats in the Wolong Reserve, China J Wildl Dis 1994 30 1 86 89 8151830 10.7589/0090-3558-30.1.86

[B4] Monson RA Stone WB Canine distemper in wild carnivores in New York N Y Fish Game J 1976 23 2 149 154

[B5] Alldinger S Baumgartner W van Moll P Orvell C In vivo and in vitro expression of canine distemper viral proteins in dogs and non-domestic carnivores Arch Virol 1993 132 3-4 421 428 10.1007/BF01309550 7691048

[B6] Gaedke K Zurbriggen A Baumgartner W In vivo and in vitro detection of canine distemper virus nucleoprotein gene with digoxigenin-labelled RNA, double-stranded DNA probes and oligonucleotides by in situ hybridization Zentralbl Veterinarmed B 1997 44 6 329 340 9283284 10.1111/j.1439-0450.1997.tb00983.x

[B7] Hoyland JA Dixon JA Berry JL Davies M Selby PL Mee AP A comparison of in situ hybridisation, reverse transcriptase-polymerase chain reaction (RT-PCR) and in situ-RT-PCR for the detection of canine distemper virus RNA in Paget's disease J Virol Methods 2003 109 2 253 259 10.1016/S0166-0934(03)00079-X 12711070

[B8] Oglesbee M Jackwood D Perrine K Axthelm M Krakowka S Rice J In vitro detection of canine distemper virus nucleic acid with a virus-specific cDNA probe by dot-blot and in situ hybridization J Virol Methods 1986 14 3-4 195 211 10.1016/0166-0934(86)90022-4 3539957

[B9] Yoshida E Iwatsuki K Miyashita N Gemma T Kai C Mikami T Molecular analysis of the nucleocapsid protein of recent isolates of canine distemper virus in Japan Vet Microbiol 1998 59 2-3 237 244 10.1016/S0378-1135(97)00194-6 9549863

[B10] Barben G Stettler M Jaggy A Vandevelde M Zurbriggen A Detection of IgM antibodies against a recombinant nucleocapsid protein of canine distemper virus in dog sera using a dot-blot assay Zentralbl Veterinarmed A 1999 46 2 115 121 10216448 10.1046/j.1439-0442.1999.00198.x

[B11] Martella V Pratelli A Cirone F Zizzo N Decaro N Tinelli A Foti M Buonavoglia C Detection and genetic characterization of canine distemper virus (CDV) from free-ranging red foxes in Italy Mol Cell Probes 2002 16 1 77 83 10.1006/mcpr.2001.0387 12005452

[B12] Mee AP Dixon JA Hoyland JA Davies M Selby PL Mawer EB Detection of canine distemper virus in 100% of Paget's disease samples by in situ-reverse transcriptase-polymerase chain reaction Bone 1998 23 2 171 175 10.1016/S8756-3282(98)00079-9 9701477

[B13] Rzezutka A Mizak B Application of N-PCR for diagnosis of distemper in dogs and fur animals Vet Microbiol 2002 88 1 95 103 10.1016/S0378-1135(02)00097-4 12119141

[B14] von Messling V Harder TC Moennig V Rautenberg P Nolte I Haas L Rapid and sensitive detection of immunoglobulin M (IgM) and IgG antibodies against canine distemper virus by a new recombinant nucleocapsid protein-based enzyme-linked immunosorbent assay J Clin Microbiol 1999 37 4 1049 1056 10074525 10.1128/jcm.37.4.1049-1056.1999 PMC88648

[B15] Grant RJ Banyard AC Barrett T Saliki JT Romero CH Real-time RT-PCR assays for the rapid and differential detection of dolphin and porpoise morbilliviruses J Virol Methods 2009 156 1-2 117 123 10.1016/j.jviromet.2008.11.008 19084557

[B16] Calderon MG Remorini P Periolo O Iglesias M Mattion N La Torre J Detection by RT-PCR and genetic characterization of canine distemper virus from vaccinated and non-vaccinated dogs in Argentina Vet Microbiol 2007 125 3-4 341 349 10.1016/j.vetmic.2007.05.020 17628358

[B17] Sidhu MS Husar W Cook SD Dowling PC Udem SA Canine distemper terminal and intergenic non-protein coding nucleotide sequences: completion of the entire CDV genome sequence Virology 1993 193 1 66 72 10.1006/viro.1993.1103 8438593

[B18] Iwatsuki K Miyashita N Yoshida E Gemma T Shin YS Mori T Hirayama N Kai C Mikami T Molecular and phylogenetic analyses of the haemagglutinin (H) proteins of field isolates of canine distemper virus from naturally infected dogs J Gen Virol 1997 78 Pt 2 373 380 9018060 10.1099/0022-1317-78-2-373

[B19] Blixenkrone-Moller M Biological properties of phocine distemper virus and canine distemper virus APMIS Suppl 1993 36 1 51 8268007

[B20] Iwatsuki K Tokiyoshi S Hirayama N Nakamura K Ohashi K Wakasa C Mikami T Kai C Antigenic differences in the H proteins of canine distemper viruses Vet Microbiol 2000 71 3-4 281 286 10.1016/S0378-1135(99)00172-8 10703710

[B21] Martella V Elia G Lucente MS Decaro N Lorusso E Banyai K Blixenkrone-Moller M Lan NT Yamaguchi R Cirone F Carmichael LE Buonavoglia C Genotyping canine distemper virus (CDV) by a hemi-nested multiplex PCR provides a rapid approach for investigation of CDV outbreaks Vet Microbiol 2007 122 1-2 32 42 10.1016/j.vetmic.2007.01.005 17275219

[B22] Zhao JJ Yan XJ Chai XL Martella V Luo GL Zhang HL Gao H Liu YX Bai X Zhang L Chen T Xu L Zhao CF Wang FX Shao XQ Wu W Cheng SP Phylogenetic analysis of the haemagglutinin gene of canine distemper virus strains detected from breeding foxes, raccoon dogs and minks in China Vet Microbiol 2010 140 1-2 34 42 10.1016/j.vetmic.2009.07.010 19647380

[B23] Blixenkrone-Moller M Sharma B Varsanyi TM Hu A Norrby E Kovamees J Sequence analysis of the genes encoding the nucleocapsid protein and phosphoprotein (P) of phocid distemper virus, and editing of the P gene transcript J Gen Virol 1992 73 Pt 4 885 893 10.1099/0022-1317-73-4-885 1634877

[B24] Visser IK Kumarev VP Orvell C de Vries P Broeders HW van de Bildt MW Groen J Teppema JS Burger MC UytdeHaag FG Osterhaus AD Comparison of two morbilliviruses isolated from seals during outbreaks of distemper in north west Europe and Siberia Arch Virol 1990 111 3-4 149 164 10.1007/BF01311050 2141248

[B25] Martella V Cirone F Elia G Lorusso E Decaro N Campolo M Desario C Lucente MS Bellacicco AL Blixenkrone-Moller M Carmichael LE Buonavoglia C Heterogeneity within the hemagglutinin genes of canine distemper virus (CDV) strains detected in Italy Vet Microbiol 2006 116 4 301 309 10.1016/j.vetmic.2006.04.019 16730927

[B26] Demeter Z Lakatos B Palade EA Kozma T Forgach P Rusvai M Genetic diversity of Hungarian canine distemper virus strains Vet Microbiol 2007 122 3-4 258 269 10.1016/j.vetmic.2007.02.001 17350769 PMC7117499

[B27] He HB Li JZ Xia XJ Yu C Fan QS Huang G Qiu W Yin Z Phylogenetic analysis of the haemagglutinin protein gene of canine distemper virus from giant panda and other animals in China Chin J Virol 2000 16 238 241 (in chinese)

[B28] Stettler M Beck K Wagner A Vandevelde M Zurbriggen A Determinants of persistence in canine distemper viruses Vet Microbiol 1997 57 1 83 93 10.1016/S0378-1135(96)01281-3 9231983

